# Graphene assisted crystallization and charge extraction for efficient and stable perovskite solar cells free of a hole-transport layer

**DOI:** 10.1039/d0ra09225h

**Published:** 2021-01-22

**Authors:** Ahmed Esmail Shalan, Mustafa K. A. Mohammed, Nagaraj Govindan

**Affiliations:** Central Metallurgical Research and Development Institute (CMRDI) P. O. Box 87 Helwan Cairo 11421 Egypt; BCMaterials, Basque Center for Materials, Applications and Nanostructures Martina Casiano, UPV/EHU Science Park, Barrio Sarriena s/n Leioa 48940 Spain; Technical Engineering College, Middle Technical University Baghdad Iraq mustafa_kareem97@yahoo.com +9647719047121; Department of Physics, Periyar University, P.G Extension Center Dharmapuri Tamil Nadu India

## Abstract

In recent times, perovskite solar cells (PSCs) have been of wide interest in solar energy research, which has ushered in a new era for photovoltaic power sources through the incredible enhancement in their power conversion efficiency (PCE). However, several serious challenges still face their high efficiency: upscaling and commercialization of the fabricated devices, including long-term stability as well as the humid environment conditions of the functional cells. To overcome these obstacles, stable graphene (G) materials with tunable electronic features have been used to assist the crystallization as well as the charge extraction inside the device configuration. Nonetheless, the hole transport layer (HTL)-free PSCs based on graphene materials exhibit unpredictable results, including a high efficiency and long-term stability even in the conditions of an ambient air atmosphere. Herein, we combine graphene materials into a mesoporous TiO_2_ electron transfer layer (ETL) to improve the coverage and crystallinity of the perovskite material and minimize charge recombination to augment both the stability and efficiency of the fabricated mixed cation PSCs in ambient air, even in the absence of a HTL. Our results demonstrate that an optimized PSC in the presence of different percentages of graphene materials displays a PCE of up to 17% in the case of a G:TiO_2_ ETL doped with 1.5% graphene. With this coverage and crystallinity amendment approach, we show hysteresis-free and stable PSCs, with less decomposition after ∼3000 h of storage under a moist ambient atmosphere. This work focuses on the originalities of the materials, expenses, and the assembling of stable and effective perovskite solar cells.

## Abstract 2

In recent times, perovskite solar cells (PSCs) have been of wide interest in solar energy research, which has ushered in a new era for photovoltaic power sources through the incredible enhancement in their power conversion efficiency (PCE).

## Graphical abstract

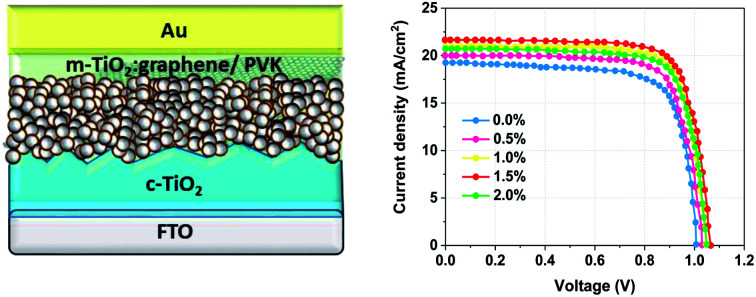

## 1. Introduction

Solution-processed perovskite solar cells (PSCs) dependent on an organometal trihalide have become promising candidates for third-generation solar cells and have been intensively focused on as an actual challenger to conventional silicon photovoltaics, attributable to their different advantages, including that they are cost-effective, easy to assemble and have high power conversion efficiencies.^1–8^

The typical structural configuration of the PSC includes an absorber perovskite (PVK) layer sandwiched between electron and hole transfer layers (ETL and HTL) that is responsible for light harvesting and charge generation.^9–15^ However, the perovskite layer can provide dual functions and serve as a sensitizer as well as a hole transport material.^16^ Besides this, TiO_2_, as a semiconducting oxide, has been extensively applied as an ETL in solar cells to deliver effective electron extraction.^17–20^ The incorporation of carbon nano-object traces, like graphene materials in the structure of semiconductor materials, are considered as one possible direction, advancing the charge collection efficiency of the fabricated devices.^21,22^ TiO_2_ has appropriate characteristics, such as a wide bandgap energy, high exciton binding energy, high light absorption and high efficiency, low cost, and acceptable electron mobility.^23^ Inappropriate conduction band alignment and low conductivity of each layer are critical disadvantages.^24^ Consequently, additional efforts have been dedicated in these directions to enhance the performance of the stabilized devices. Graphene and other carbon nanomaterials are promising candidates for this objective.^25–27^ A monolayer of graphene and/or its derivatives with a honeycomb lattice have caused much fascination to engage them in solar cells for the reason that they serve as a rapid charge funnel.^28–30^

Predominantly, graphene materials can improve the conductivity and deliver a better band alignment between the different layers. Also, graphene has been applied as a dopant or interlayer in PSCs, in which it can improve the morphological and crystalline features of the PVK material, afford a proficient charge-extraction pathway and support electron collection from the PVK film to the ETL.^31^ Different researchers have studied the addition of graphene materials to different ETLs to augment charge injection and reduce the recombination process.^32,33^ However, the effect of graphene on the coverage and crystallinity of the perovskite layer as well as the long-term stability of PSCs have not been well investigated. Therefore, it is essential to additionally discover the positive effect of graphene materials in the structure, efficiency and stability of the fabricated cells.

Herein, we assembled mixed cation HTL-free PSCs in ambient air based on both bare TiO_2_ and TiO_2_ doped with different molar ratios of graphene materials. Graphene materials are incorporated into the mesoporous TiO_2_ ETL to enhance the coverage and crystallinity of the perovskite material and minimize charge recombination to boost both the stability and efficiency of the fabricated mixed cation PSCs in ambient air, even in the absence of a HTL. The gained PCE of the assembled PSCs is improved from 12.48% to reach almost 16.75% with a smaller hysteresis effect after the graphene addition to the TiO_2_ ETL. With this coverage and crystallinity amendment approach, the devices demonstrate less decomposition after ∼3000 h of storing in a moist ambient environment. This study affords a practical way to acquire stable and efficient mixed cation PSCs in ambient air and depicts the essential role of graphene in modifying the interfacial contact between TiO_2_ and PVK, as well as assembling stable and effective perovskite solar cells.

## 2. Experimental

### 2.1. Chemicals and reagents

Unless otherwise stated, all the materials were purchased from Merck and used as received. *N*,*N*-Dimethylformamide (DMF, 99.8%), formamidinium iodide (FAI, 99%), and methylammonium bromide (MABr, 99%) were provided by Dyesol. Isopropanol (IPA, 99.5%), lead iodide (PbI_2_), titanium diisopropoxide (TAA), and m-TiO_2_ were ordered from Sigma-Aldrich. Graphene powder (purity ∼99%, sheet thickness ≤10 nm) was provided by Alibaba.

### 2.2. Device fabrication

All the operations were performed under ambient air conditions and without any control method. The patterned-FTO glasses were successively cleaned using a detergent solution, deionized water, IPA, and ethanol in an ultrasonic bath for 5 min (every step). The FTO substrates were dried in an oven at 80 °C for 20 min and treated with an ultraviolet (UV)-ozone cleaner for 15 min before use. A compact-TiO_2_ (c-TiO_2_) (0.18 M TAA in ethanol) was spin-coated at 4000 rpm for 45 s and sintered at 300 °C for 10 min. Then, the mixtures of G/TiO_2_ (0.0%, 0.5%, 1.0%, 1.5%, and 2.0%) were spun at 5000 rpm for 35 seconds by employing a TiO_2_ paste dispersed in ethanol at 1 : 6 and baked at 425 °C for 15 min. The ETLs were immersed in a 0.025 M titanium tetrachloride aqueous solution at 70 °C for 20 min, then dried with N_2_ gas and again baked at 425 °C for 20 min. For the mixed cation perovskite, 0.458 g of lead iodide (PbI_2_) and 0.065 g of DMSO were added to 0.5 g of DMF and mixed for 1 h at ambient conditions. The FAI : MABr mixture (0.05 g : 0.005 g) was dispersed in 1 mL of the IPA solvent. The PbI_2_ precursor was spun onto the G:TiO_2_ ETL at 4000 rpm for 20 s. FAI:MABr mixtures were deposited on top of the PbI_2_ film at 5000 rpm for 40 s and then annealed on the hotplate at 120 °C for 15 min. Finally, 80 nm of a Au counter electrode was evaporated to complete the HTL-free device fabrication.

### 2.3. Characterization

The morphological properties of the PVK films were measured by scanning electron microscopy (SEM, TESCAN). The crystalline nature of the perovskite was measured using XRD analysis (Bruker, D8 Advance). The optical characteristics of perovskites were measured *via* photoluminescence (PL) spectroscopy (Varian Cary Eclipse Fluorescence) and UV-visible spectroscopy (Ocean Optics). Current density–voltage (*J*–*V*) characteristics of the devices were determined by a Keithley Model 2400 under 100 mW cm^−2^ (AM 1.5G one sun) illumination. The PSCs have an active area of 0.07 cm^2^. The charge generation and collection were monitored by an IPCE system (BunkoukeikiCEP-1500). The Nyquist plots were measured *via* electrochemical impedance spectroscopy (EIS, electrochemical workstation, Zennium 400147). The time-resolved PL (TRPL) was recorded at 760 nm employing excitation with a 478 nm light pulse from Delta Flex Fluorescence Lifetime System (Horiba Scientific Com.).

## 3. Results and discussion

**Fig. 1:**
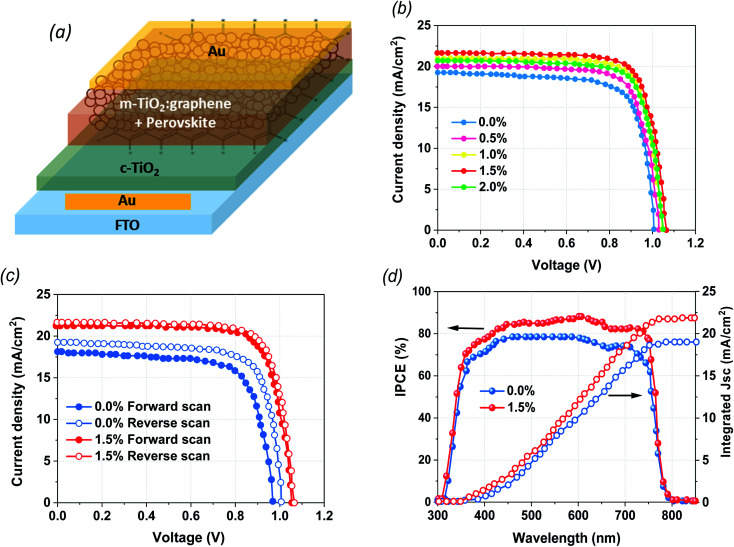
(a) Schematic illustration of the architecture of the devices fabricated in this study. (b) *J*–*V* plots of PSCs with different amounts of graphene (0.0%, 0.5%, 1.0%, 1.5%, and 2.0%) in the G/TiO_2_ layer. (c) Hysteresis effect of PSCs with pure TiO_2_ and TiO_2_ containing 1.5% graphene. (d) EQE spectra of PSCs with and without graphene doping.

The structural configurations of the fabricated devices in the current study are illustrated in Fig. 1a. The mixed cation perovskite (PVK) layers were grown directly on the mixtures of G:TiO_2_ (0.0%, 0.5%, 1.0%, 1.5%, and 2.0%) photoelectrode films that work as ETLs *via* a two-step spin-coating deposition technique in ambient conditions. The architecture of FTO/c-TiO_2_/m-TiO_2_:graphene/perovskite/Au demonstrates the impact of graphene materials on the loss of a hole transport layer (HTL) in PSCs free of HTLs. After that, the devices were completed with thermally evaporated Au back contacts. In the current work, the essential role of graphene materials is to modify the crystallization of the perovskite film to improve the surface coverage as well as the interfacial contact between G/TiO_2_ and the PVK layer. Consequently, graphene materials have a valuable impact on lessening the instability of the PVK on top of the G:TiO_2_ film. To detect the efficiency of the fabricated devices, the photovoltaic (PV) parameters were checked through *I*–*V* measurements. The *J*–*V* curves of the optimized PSCs with altered molar ratios of graphene (0.0%, 0.5%, 1.0%, 1.5%, and 2.0%) in G:TiO_2_ as the ETLs were measured at reverse scan conditions under air mass 1.5 global (AM 1.5 G) conditions (Fig. 1b). The device with bare TiO_2_ and without the addition of graphene (reference cells) displays a PCE of 12.48%, whereas the addition of graphene materials to the composition of TiO_2_ with different molar ratios of G:TiO_2_ results in an augmentation in the overall efficiency. We find that G:TiO_2_ samples with 1.5% graphene are the optimal composition for PV performance. A maximum PCE of 16.75%, with *V*_oc_ of 1.065 V, *J*_sc_ of 21.71 mA cm^−2^, and FF of 73.47% were detected for that composition. This improvement is accredited to the enhanced *V*_oc_ and *J*_sc_ because of the higher conductivity and lower trap states in the G:TiO_2_. The addition of graphene weakens the reaction of MAI and TiO_2_, which could keep the long lifetime of the TiO_2_ layer. Besides this, the main characterization methods were carried out on 0.0% G:TiO_2_ and 1.5% G:TiO_2_ for the comparative study. We considered the hysteresis effect of the examined cells at the forward and reverse scans, which is obviously reduced in the case of 1.5% G:TiO_2_ based devices (Fig. 1c). The low hysteresis effect indicated for 1.5% G:TiO2 can be accredited to the better charge transfer and reduction of recombination because of a higher conductivity and lower trap states in the G:TiO_2_ layer. In addition, the validity of *J*_sc_ findings for the 0.0% G:TiO_2_ and 1.5% G:TiO_2_ samples was confirmed *via* external quantum efficiency measurements (Fig. 1d). The integrated *J*_sc_ values derived from the EQE data were 19.10 mA cm^−2^ and 21.60 mA cm^−2^, respectively, for the 0.0% G:TiO2 and 1.5% G:TiO_2_ PSCs, which are in decent alignment with the *J*_sc_ values gained from the *J*–*V* curves (Fig. 1b). Besides this, all the photovoltaic parameters of the fabricated HTL-free PSCs with a diverse amount of graphene (0.0%, 0.5%, 1.0%, 1.5%, and 2.0%) in G:TiO2 layer are listed in Table 1.

**Table 1:** Photovoltaic parameters of the fabricated HTL-free PSCs with different amounts of graphene

Device		*V*_oc_ a (V)	*J*_sc_ b (mA cm^−2^)	FF^c^ (%)	PCE^d^ (%)
0.0%	Average	0.975	18.67	63.49	11.55
	Best	1.003	19.25	64.65	12.48
0.5%	Average	1.004	19.18	65.32	12.56
	Best	1.024	20.01	66.29	13.58
1.0%	Average	1.012	20.02	68.27	13.83
	Best	1.036	20.92	69.87	15.14
1.5%	Average	1.031	20.45	71.71	15.19
	Best	1.065	21.71	73.47	16.75
2.0%	Average	1.012	19.85	67.13	13.48
	Best	1.044	20.70	68.45	14.79

a *V*_oc_: open-circuit voltage.

b *J*sc: short-circuit current density.

c FF: fill factor.

d PCE: power conversion efficiency.

**Fig. 2:**
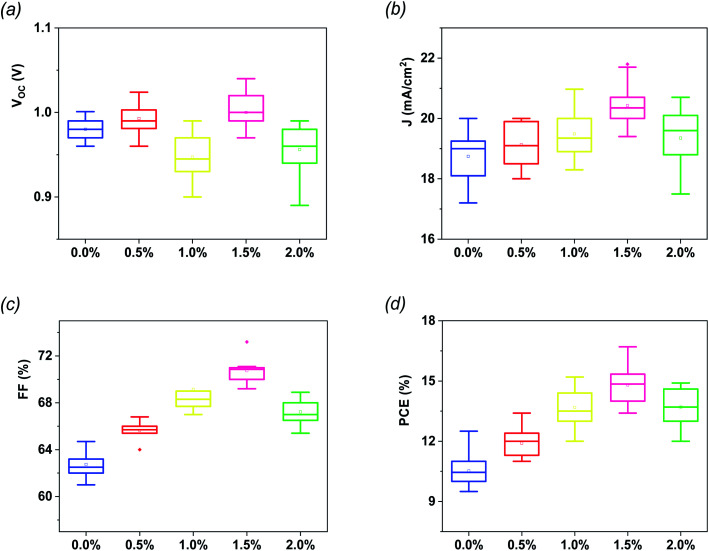
Statistical histograms of photovoltaic parameters for different PSCs (a) *V*_oc_, (b) *J*_sc_, (c) FF and (d) PCE. Every box shows the parameter distribution of 20 devices.

The statistical analysis as well as the average photovoltaic parameters of 20 fabricated devices for each composition of G:TiO_2_ are shown in Fig. 2. The corresponding box chart plots of *J*_sc_, *V*_oc_, and FF for the different amounts of graphene (0.0%, 0.5%, 1.0%, 1.5%, and 2.0%) in the G:TiO_2_ layer can be found in Fig. 2(a–d), representing a superb reproducibility with a limited variation. The highest parameters were detected for the device using TiO_2_ containing 1.5% graphene as an ETL, principally because of the highest *J*_sc_ value that is attributed to the greatest effectual interfacial charge transfer and the lowest carrier recombination, as affirmed from EIS measurements (see Fig. 4c).^34,35^

**Fig. 3:**
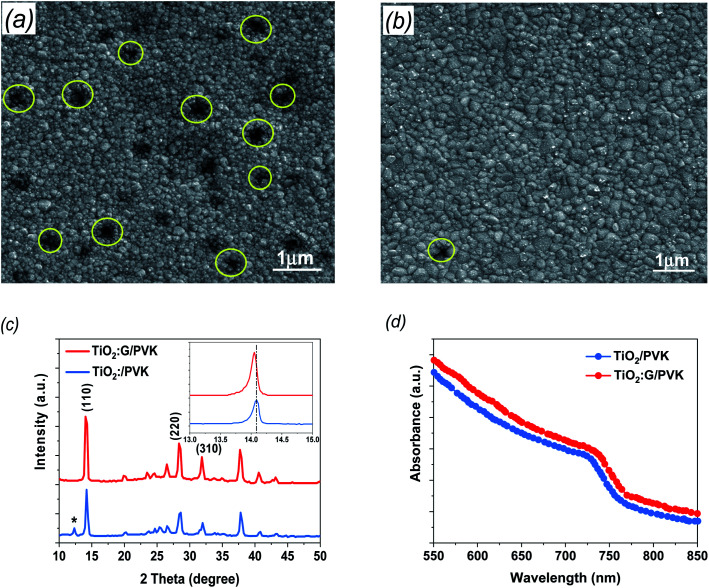
(a) FESEM image of perovskite deposited on a pure TiO_2_ layer. (b) Perovskite on a G:TiO_2_ layer. (c) XRD patterns of perovskites. The asterisk indicates the reflection of PbI_2_. Inset: zoomed-in view of the peak at 14° of the perovskites. (d) Absorption spectra of perovskite films deposited on pure and modified TiO_2_ layers.

To gain further information about the prepared G:TiO_2_ applied in the device structure, different characterization techniques were utilized. The surface morphologies of the perovskite layer on bare TiO_2_ as well as on G:TiO_2_ films were checked *via* FESEM analysis and are illustrated in Fig. 3 (a and b), respectively. The SEM image of perovskite over 0.0% G:TiO_2_ film displays different pinholes and drops in the covering process (Fig. 3a), while after adding graphene to the composition of the TiO_2_, the surface coverage as well as the crystallinity of the deposited perovskite film were enhanced due to the higher conductivity gained from the existence of graphene materials (Fig. 3b). The results obtained from the SEM image of perovskite over the 1.5% G:TiO_2_ film confirm the importance of graphene doping inside the ETL to gain a uniform layer with no major alterations in the surface morphology. Besides, the perovskite layer deposited on the 1.5% G:TiO_2_ film has a greater average grain size than that on the 0.0% G/TiO_2_ film, elucidating the higher value of *V*_oc_ and a lower hysteresis in 1.5% G:TiO_2_ PSCs. Furthermore, the XRD patterns of the resulting perovskite films (Fig. 3c) yielded identical crystal structures with (110), (220), and (310) diffraction peaks at 14.07°, 28.3°, and 31.7°. The peak at 14.07° in the 0.0% G:TiO_2_/perovskite film corresponds to the PbI_2_ phase correlated with the decomposition of a small amount of perovskite during thermal annealing.^36^ On the other hand, no peak of PbI_2_ could be detected in the XRD of the 1.5% G:TiO_2_/perovskite film. Also, the peak intensities of the 1.5% G:TiO_2_/perovskite film designate a considerable development compared to those of the 0.0% G:TiO_2_/perovskite film, especially for the (110) peak, indicating that the addition of graphene could augment the crystallinity of mixed perovskite.^37^

To elucidate the optical properties of the deposited perovskite layer over TiO_2_ and TiO_2_ containing 1.5% graphene as an ETL, we checked these data *via* UV-visible spectrophotometry (Fig. 3d). The absorbance of both samples established a comparable spectrum in the visible region with an onset at 780 nm, which slightly increased after the addition of 1.5% graphene doping and is consistent with a previous report.^38^ In addition, the acquired results display that the perovskite films deposited over TiO_2_ containing 1.5% graphene can harvest more light, which should be satisfactory to advancing the performance of the fabricated PSCs.

**Fig. 4:**
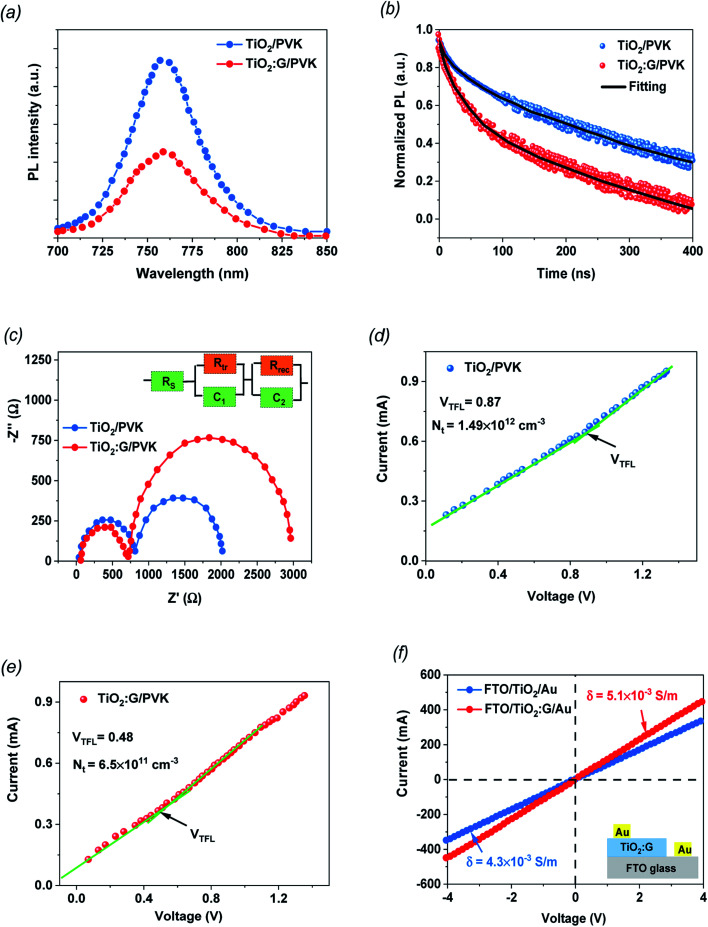
(a) Steady-state PL spectra. (b) TRPL spectra. (c) Nyquist plots with charge-transfer resistances of 812 Ω and 718 Ω for pure and doped devices, respectively, with the corresponding recombination resistances of 2018 Ω and 2962 Ω. Dark current–voltage curves of the electron-only devices for (d) FTO/TiO_2_/PVK//PCBM/Au and (e) FTO/G:TiO_2_/PVK/PCBM/Au. (f) *I*–*V* curves of the devices with an FTO/ETL/Au structure for conductivity measurements.

PL spectroscopy was used to check the charge-extraction properties of the fabricated PSC devices based on PVK deposited on TiO_2_ and TiO_2_ containing 1.5% graphene as the ETL films. The steady-state PL (Fig. 4a) was measured for both samples to evaluate the influence of PL quenching of the PVK. The perovskite layer on the 1.5% G:TiO_2_ film displays a noteworthy PL-quenching effect that confirms the improved behavior in charge collection because of the lower surface defects at the interface, supported by the graphene doping. TRPL analysis was established to further investigate the photo-induced charges dynamics, including charge extraction and recombination. Fig. 4b demonstrates the PL transient profiles of PVK deposited on TiO_2_ and TiO_2_ containing 1.5% graphene as the ETL films and displays the average recombination lifetime (*τ*_ave_) that can be checked *via* a three-component exponential decay function.^42^ The fitted time coefficients (*τ*_1_, *τ*_2_ and *τ*_3_) with the corresponding relative amplitudes (*A*_1_, *A*_2_ and *A*_3_) based on the three-component exponential decay function for TRPL spectra of the PSCs with and without graphene are summarized on Table 2. The optimized PSC with TiO_2_ containing 1.5% graphene shows a significant drop in the PL decay time data compared with that of the control PSC based on TiO_2_ without graphene doping. Besides, the *τ*_ave_ is reduced from 137.32 ns to 105.43 ns after graphene incorporation, setting an improved electron injection from the PVK layer into the TiO_2_ containing 1.5% graphene. Furthermore, the Nyquist measurements were checked by EIS studies for the fabricated PSC devices based on PVK deposited on TiO_2_ and TiO_2_ containing 1.5% graphene as the ETL films to additionally recognize to the role of the graphene additive (Fig. 4c). It is clear that the arc of the PSC with the TiO_2_ containing 1.5% graphene film is smaller than that of PSC based on the pure TiO_2_ film, indicating that the hybrid photoelectrode of graphene and TiO_2_ suppresses the charge-transport resistance (*R*_tr_). Nyquist plots show charge-transfer resistances of 812 Ω and 718 Ω for the devices based TiO_2_ and TiO_2_ containing 1.5% graphene as an ETL, respectively, with corresponding recombination resistances of 2018 and 2962 Ω for both devices, respectively. It is evident that the cell with a photoelectrode doped with graphene exposes a smaller charge-transport resistance and higher recombination resistances, signifying a fast charge transfer ability and a lower recombination rate compared to those of the control cell, as evidenced by the higher *J*_sc_ values.^39,40^ Additionally, the inset in Fig. 4c discusses the equivalent circuits of the PSCs. Furthermore, the space-charge-limited current (SCLC) model was used to recognize the mechanism of conductivity enhancement for TiO_2_ and G:TiO_2_ ETLs with an electron only cell architecture, and the obtained *J*–*V* plots are described in Fig. 4(d and e). Besides, the *I*–*V* curves are linear in the low voltage region and then the current discloses a sharp increment after a certain voltage *i.e.*, trap-filled limit voltages (*V*_TFL_) because the trap states have been filled. Correspondingly, the *V*_TFL_ values of the pure TiO_2_ film and the G:TiO_2_ films are 0.87 and 0.48 V, respectively. The trap-state density (*n*_trap_) of G:TiO_2_ (6.50 × 10^11^ cm^−3^) is lower than that of pure TiO_2_ (1.49 × 10^12^ cm^−3^). Such a higher electron mobility and lower trap density of the G:TiO_2_ film is favorable for an efficient forward electron flow and as a consequence the photovoltaic performance of the perovskite solar cells has been developed.

**Table 2:** Fitting parameters using a three-component exponential decay function for the TRPL spectra of PSCs with and without graphene

Type	*A*_1_	*τ*_1_ (ns)	*A*_2_	*τ*_2_ (ns)	*A*_3_	*τ*_3_ (ns)	*τ*_ave_ (ns)
TiO_2_/PVK	0.38	6.67	0.32	28.32	0.48	155.03	137.32
G:TiO_2_/PVK	0.52	5.92	0.42	22.45	0.21	141.89	105.43

Moreover, the *I*–*V* measurements were checked through the sandwich structures of FTO/TiO_2_/Au and FTO/G:TiO_2_/Au to examine the change in electrical properties of the TiO_2_ ETL through the addition of graphene materials. The bias potential between the FTO and Au electrodes continuously increased from −1 V to 1 V and the thicknesses of the ETL are the same in the cases of TiO_2_ and G:TiO_2_. The current–voltage (*I*–*V*) plots recorded in the dark for various ETLs are demonstrated in Fig. 4f. The augmentation in the current was detected for the G:TiO_2_ ETL. The acquired curves illustrate that the conductivity (*σ*) of the different ETLs was found to be 4.30 × 10^−5^ and 5.10 × 10^−5^ S m^−1^ for FTO/TiO_2_/Au and FTO/G:TiO_2_/Au, respectively, showing a higher conductivity for G:TiO_2_ samples compared to that of the bare TiO_2_.^41^

**Fig. 5:**
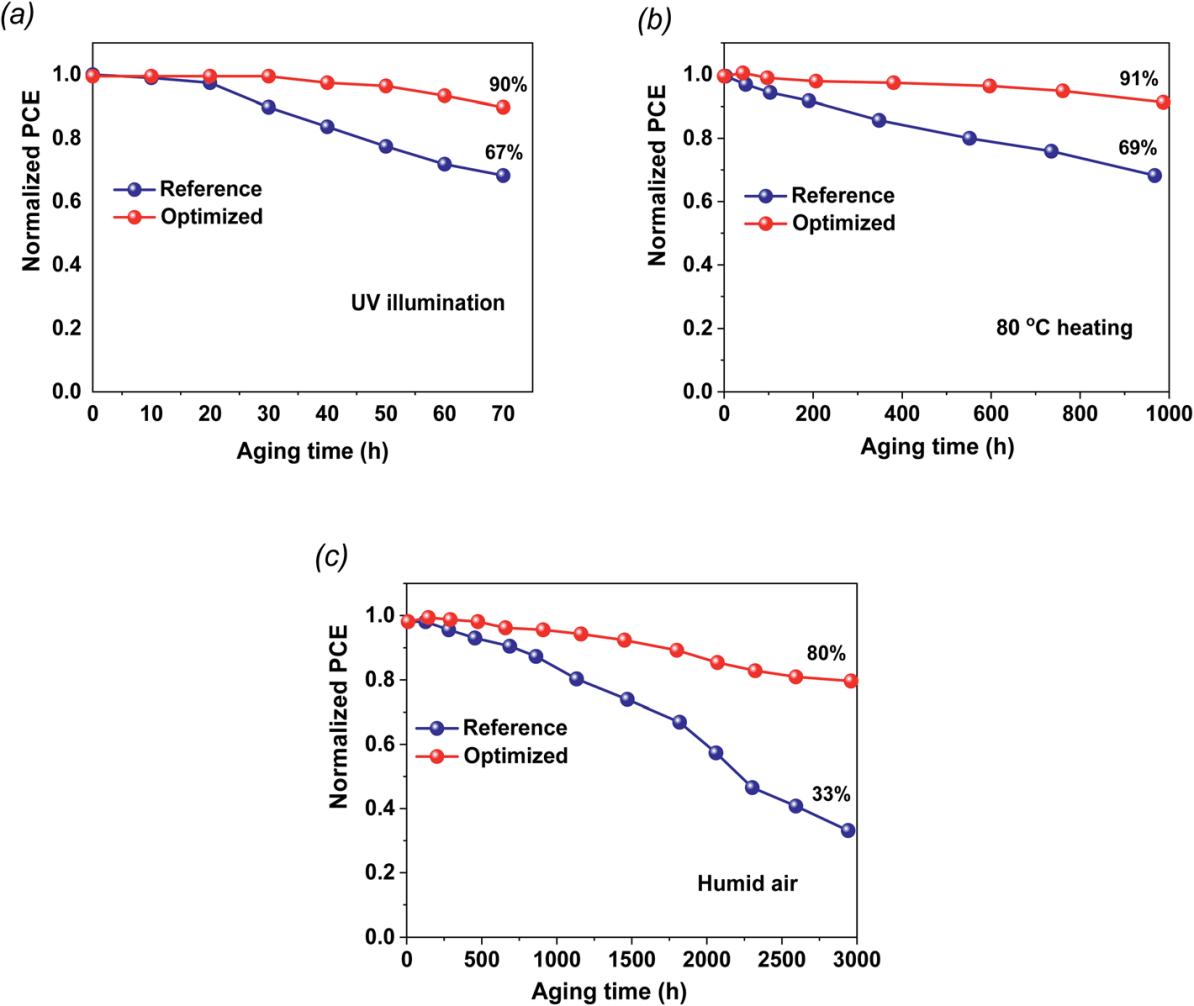
Stability performance of PSCs under various conditions. All the devices are unencapsulated and the reference devices are compared with 1.5% graphene-containing devices. (a) Stability test of the perovskite device on TiO_2_ and on G:TiO_2_ ETLs under continuous UV light illumination at room temperature. (b) Devices kept at 80 °C under a nitrogen atmosphere. (c) Devices in ambient air with a RH of 25–35% at room temperature.

The stability of the fabricated cells in ambient conditions and during thermal annealing is a fundamental challenge for dedicated commercialization. The shelf-life stabilities of the FTO/TiO_2_/PVK/Au and FTO/G:TiO_2_/PVK/Au devices were checked without any encapsulation (Fig. 5). The stability test results of the perovskite devices based on TiO_2_ and on G:TiO_2_ ETLs under continuous UV light illumination at room temperature are found in Fig. 5a. The devices that contain graphene doped TiO_2_ and bare TiO_2_ as the ETLs retained 90 and 87%, respectively, of the initial efficiency results. Besides, the 1.5% G:TiO_2_ device retained 80% of its initial PCE, which designates a superior reliability comparative to that of the 0.0% G:TiO_2_ device (∼33% of its initial PCE). Additionally, PCE of the unencapsulated device retained more than 91% after thermal annealing at 80 °C for 1000 h under ambient conditions, demonstrating the superior thermal stability of the 1.5% G:TiO_2_ doped device compared to that of the bare TiO_2_ device (Fig. 5b). The devices were stored in wet conditions (∼30% RH) and the PV characteristics were measured over 3000 h (Fig. 5c). The improved stability of the 1.5% G:TiO_2_ device could be accredited to improved features of the ETL and lower trap states in the graphene-doped TiO_2_. The devices based on the G:TiO_2_ ETL display an outstanding performance and sensible stability that propose the awesome candidacy of G:TiO_2_ ETL for utilization in perovskite solar cells.

## 4. Conclusion

In summary, graphene material was doped into TiO_2_ with different molar ratios to obtain reliable ETLs for fabricating high-performance and long-term stable HTL-free PSCs. The best efficiency of 16.75% was achieved with a lower hysteresis effect by using 1.5% G:TiO_2_ as the ETL. Graphene materials contribute to a better interfacial contact between the TiO_2_ ETL and perovskite and permit perovskite to grow with bigger grains and a pinhole-free surface. Several techniques, including XRD, absorbance, FESEM, PL and cell parameters, were applied to give detailed studies and characterize the fabricated materials and devices. Additionally, the fabricated PSCs based on G:TiO_2_ ETL indicate a long-term stability in the ambient environment compared to that of the pristine devices. The current study supports that including graphene in the TiO_2_ structure is a feasible method to achieve high-quality mixed cation perovskite solar cells in ambient air for outstanding HTL-free PSCs.

## Conflicts of interest

The authors declare that they have no conflicts of interest.

## Supplementary Material
